# Phosphorylation Sites in Protein Kinases and Phosphatases Regulated by Formyl Peptide Receptor 2 Signaling

**DOI:** 10.3390/ijms21113818

**Published:** 2020-05-27

**Authors:** Maria Carmela Annunziata, Melania Parisi, Gabriella Esposito, Gabriella Fabbrocini, Rosario Ammendola, Fabio Cattaneo

**Affiliations:** 1 Department of Clinical Medicine and Surgery, School of Medicine, University of Naples Federico II, Via S. Pansini 5, 80131 Naples, Italy; marica.annunziata@unina.it (M.C.A.); melania.parisi@unina.it (M.P.); gafabbro@unina.it (G.F.); 2 Department of Molecular Medicine and Medical Biotechnology, School of Medicine, University of Naples Federico II, Via S. Pansini 5, 80131 Naples, Italy; gabriella.esposito@unina.it (G.E.); rosario.ammendola@unina.it (R.A.)

**Keywords:** FPR2, cell signaling, phospho-sites, PKN2, PRP4, MARK2, PAK4, STK10, MAP2K2, PPP1R14A

## Abstract

FPR1, FPR2, and FPR3 are members of Formyl Peptides Receptors (FPRs) family belonging to the GPCR superfamily. FPR2 is a low affinity receptor for formyl peptides and it is considered the most promiscuous member of this family. Intracellular signaling cascades triggered by FPRs include the activation of different protein kinases and phosphatase, as well as tyrosine kinase receptors transactivation. Protein kinases and phosphatases act coordinately and any impairment of their activation or regulation represents one of the most common causes of several human diseases. Several phospho-sites has been identified in protein kinases and phosphatases, whose role may be to expand the repertoire of molecular mechanisms of regulation or may be necessary for fine-tuning of switch properties. We previously performed a phospho-proteomic analysis in FPR2-stimulated cells that revealed, among other things, not yet identified phospho-sites on six protein kinases and one protein phosphatase. Herein, we discuss on the selective phosphorylation of Serine/Threonine-protein kinase N2, Serine/Threonine-protein kinase PRP4 homolog, Serine/Threonine-protein kinase MARK2, Serine/Threonine-protein kinase PAK4, Serine/Threonine-protein kinase 10, Dual specificity mitogen-activated protein kinase kinase 2, and Protein phosphatase 1 regulatory subunit 14A, triggered by FPR2 stimulation. We also describe the putative FPR2-dependent signaling cascades upstream to these specific phospho-sites.

## 1. Introduction

Post-translational modifications (PTMs) regulate many proteins functions thanks to the addition of a functional chemical group. Phosphorylation is the most important PTMs in eukaryotic cells as it plays a critical role in regulating many eukaryotic cellular processes, including metabolism, cell cycle progression, cellular proliferation, survival, migration and differentiation [1]. Phosphorylation is synergistically balanced by the action of protein kinases (PK) and protein phosphatases (PP), which are crucial signaling mediators that act coordinately to drive signal responses [2,3]. In human cells, 518 PKs catalyze about 100,000 phosphorylation events [4,5] and more than 200,000 phosphorylation sites are currently registered in PhosphoSitePlus databases [6]. On the other hand, about 160 PPs specifically regulate the phosphorylation status of target substrates [7]. Any impairment of PKs and/or PPs activation, as well as any deviation from their balance, represents one of the most common causes of several human diseases. In human cells, phosphorylation mainly occurs at serine and threonine residues (Ser/Thr) and at a lower level at tyrosine (Tyr) residues. However, Tyr phosphorylation represents a prerequisite in cellular signaling mechanisms triggered by tyrosine kinase receptors (TKR) [8,9]. Despite a significant number of phosphorylation sites in PKs and PPs has been identified, their regulatory role and the connections between each phosphorylation site and the corresponding upstream PK remain largely unexplored. Specific phospho-sites may control different functions and the multiple phosphorylation sites observed in many PKs and PPs may play the role to expand the repertoire of molecular mechanisms of regulation or may be necessary for fine-tuning of switch properties [10,11].

Formyl Peptides Receptors (FPRs) belong to the GPCR superfamily and include three members (FPR1, FPR2 and FPR3) that recognize formyl peptides and/or other ligands with different range of affinity [12,13]. FPR2 is a low affinity receptor for formyl peptides and is considered the most promiscuous member of FPRs, since it is able to recognize a broad variety of endogenous or exogenous ligands of lipidic or proteic nature, as well as formylated and not-formylated peptides [12,14]. FPR2 can modulate both pro- and anti-inflammatory response, depending on the nature of its agonist and on different recognition sites of the receptor [12], and shows a nuclear localization sequence (NLS) in the third cytoplasmic loop making it a receptor functionally expressed onto nuclear membrane of CaLu-6 epithelial cells [15].

FPRs were firstly identified on myeloid cell membrane, but subsequently their expression was also observed in neuronal, endothelial and epithelial cells [16]. Their activation elicits immune innate and inflammatory responses, as well as cell migration, proliferation, superoxide generation and contribute to several physio-pathological processes [13].

Intracellular signaling cascades triggered by FPRs include the activation of different kinases and phosphatases, such as protein kinase C (PKC) isoforms, phosphoinositide 3-kinase (PI3K), protein kinase B (Akt), mitogen-activated protein kinase (MAPK), p38MAPK, PTEN, PTP-PEST, and DUSP3/VHR [12,16,17,18,19,20], as well the phosphorylation and membrane translocation of p47^phox^ and p67^phox^ which are required for NADPH oxidase-dependent reactive oxygen species (ROS) generation [21,22]. Furthermore, FPR1 and FPR2 induce ROS-dependent TKR transactivation, as well as the phosphorylation and nuclear translocation of regulatory transcriptional factors [12,23,24,25,26].

These signaling cascades regulate several cellular functions and their impairment significantly contributes to the development of human diseases. In fact, FPRs are functionally expressed in cells and tissues of central and peripheral nervous system, where they play a key role in the development of neurodegenerative diseases [17,27], such as Alzheimer’s disease [28] and Parkinson’s disease [27,29]. Moreover, several of structurally distinct FPRs ligands result implicated in the pathophysiology of HIV infection, amyloidosis, prion disease, stroke/ischemia reperfusion injury and stomach ulcers [30,31]. FPRs may play detrimental or protective roles in cancer, depending on the cell type. In epithelial or neuronal cells FPRs expression and/or stimulation contributes to tumor proliferation, invasion and dissemination, predicting a poor prognosis in neuroblastoma [32], in ovarian cancer [33], in lung cancer [34], and in pancreatic cancer [35]. On the other hand, FPR1 exerts protective role in breast and colorectal cancer, where its functional expression seems required to achieve effective therapy [36,37].

We previously performed a phospho-proteomic analysis in FPR2-stimulated cells to shed light on intracellular signaling cascades triggered by this receptor. To this aim, CaLu-6 cells were serum-starved for 24 h and then stimulated for 5 min with the WKYMVm peptide, a selective FPR2 agonist [38]. We identified 290 differentially phosphorylated proteins and among these we detected phosphorylation sites belonging to six PKs and one PP dependent on FPR2 signaling. Herein, we discuss about the selective phosphorylation at Thr958 residue of Serine/Threonine-protein kinase N2, at Tyr849 residue of Serine/Threonine-protein kinase PRP4 homolog, at Ser486 residue of Serine/Threonine-protein kinase MARK2, at Ser181 residue of Serine/Threonine-protein kinase PAK4, at Ser438 and Thr952 residues of Serine/Threonine-protein kinase 10, at Thr394 residue of Dual specificity mitogen-activated protein kinase kinase 2, and at Ser16 and Ser26 residues of Protein phosphatase 1 regulatory subunit 14A. We also speculate on the putative signaling cascades upstream to these site-specific phosphorylations and their contribution in cellular functions.

## 2. Serine/Threonine-Protein Kinase N2 (PKN2; UniProt: Q16513)

Protein kinase C-related kinases (PKN/PRK) belong to a subfamily of AGC serine/threonine-protein kinases [39,40,41]. Thus far, three different isoforms widely expressed in mammals and eukaryotic organisms, namely PKN1 (PKNα/PRK1/PAK-1), PKN2 (PKNγ/PRK2/PAK-2) and PKN3 (PKNβ/PRK3), have been identified [42]. They share the same overall structure, closely related to the PKC family members in the COOH-terminal catalytic domain, with the greatest variation within the NH_2_-terminal GTPase-binding region. The central domain shows a weak homology to the calcium-dependent phospholipid binding C2 domain of PKC (HR2/C2) and contains an auto-inhibitory region [43,44,45]. The GTPase-binding domain, also called HR1 (homology region 1) plays a key role in PKNs regulation [44,46,47] and contains three tandem repeats (HR1a-c), which form independent GTPase-binding modules. HR1a and HR1b bind to Rho GTPases in PKN1/2, whereas RhoA preferentially binds PKN2 [45,46,48,49]. The binding to Rho induces a conformational change in PKNs, which is required for their association with Phosphoinositide-Dependent Kinase-1 (PDK1) that in turn, phosphorylates PKNs in the activation loop, thereby increasing the kinase activity [50,51]. The HR2/C2-like domain is involved in the lipids-mediated activation of PKNs or in their targeting on the membrane [45,47]. The COOH-terminal region of the three isoforms contains the Ser/Thr kinase domain [50] that in PKN2, is separated from the regulatory NH_2_-terminus by a hinge region. The 3′-end of the COOH-terminal region of PKN1 and PKN2 is also involved in the binding to RhoA [52]. PKN2 structure includes four conserved domains (C1–C4) interspersed by five variable domains (V1–V5) [53]. Two highly conserved motifs are present in the V5 domains of PKC/PRKs/PKNs members, namely a turn phosphate (corresponding to the Thr958 residue in PKN2) and a hydrophobic motif (Phe-X-X-Phe-Ser/Thr(P)-Phe/Tyr) [54,55]. The phosphorylation of these two motifs is required for the maturation process that actives the catalytically inactive PKNs [56] and may provide docking sites for targeting with other members of PKC/PRKs/PKNs family and for protein–protein interactions [54,55]. In PKN2, site-direct mutagenesis of Thr958 residue in Ala results in a kinase-dead mutant [53,57].

PKN1 and PKN2 kinase activity is regulated by mTORC2-dependent phosphorylation of the turn phosphate motif [58]. The activation of mTORC2 is matter of debate. It is activated by signals from growth factors through molecular mechanisms that might involve the binding of its component SIN1 with phosphatidylinositol (3,4,5)-trisphosphate (PIP3) [59,60]. TKR activation induces PI3K activity, resulting in increased PIP3. β- and α-adrenergic signaling through GPCR also modulates mTORC2 [61,62] and Gβγ subunits of chemoattractant receptors activate mTORC2 [63,64]. However, it is likely that mTORC2 preserves a basal level of activation, which is further enhanced upon external stimuli. These observations place PKNs downstream of PI3K, PDK1, and mTOR suggesting that PKNs may receive multiple PI3K-dependent inputs.

The biological responses downstream to the interaction of chemoattractants with FPRs are regulated by Ras and Rho GTPases family, which induce the activation of PI3K, AKT and ERK1/2 [65,66,67]. PI3K catalyze PIP3 synthesis which is required for mTORC2 activation and, in turn, for PKN2 phosphorylation in the turn phosphate motif. PI3K is also the kinase upstream of PDK1 that phosphorylates PKN2 in the activation loop [50,68] even though, at least for PKN2, activation loop phosphorylation by PDK1 is not a critical point of regulation [50].

**Figure 1:**
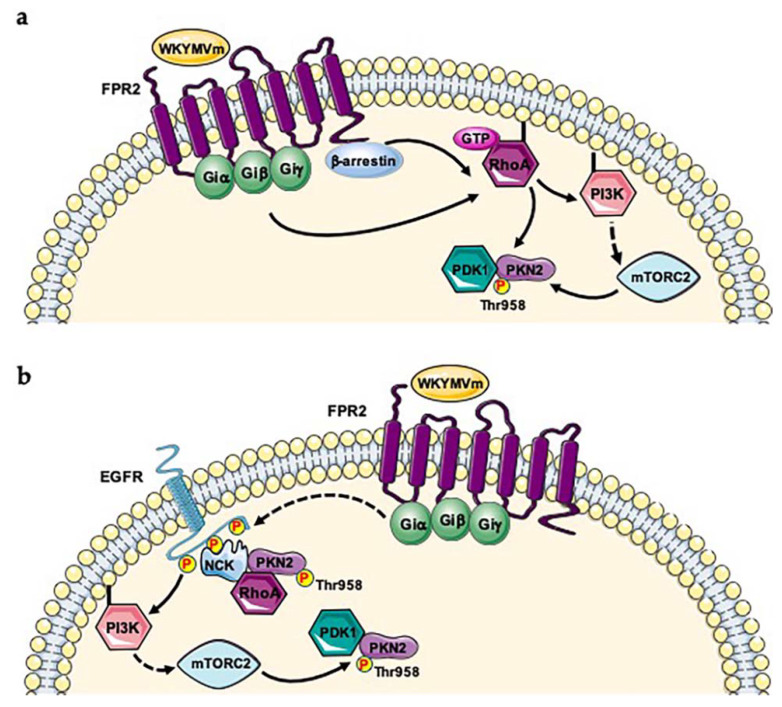
FPR2 signaling induces Thr958 phosphorylation of PKN2. Two proposed mechanisms for PKN2 activation. (**a**) FPR2 stimulation induces Rho signaling allowing a conformational change in PKN2 and the phosphorylation in the activation loop by PDK1. FPR2 triggers Rho-dependent PI3K activation both in β-arrestin-dependent or -independent manner. PI3K activates mTORC2 which phosphorylates PKN2 at Thr958 residue. (**b**) Phospho-tyrosines of trans-phosphorylated EGFR provide docking sites for NCK binding and the SH3 domains of the adapter protein bind PKN2 and Rho. Rho induces a conformational change of PKN2 which is required for binding to PDK1 and the phosphorylation in the activation loop. EGFR-induced PI3K activity triggers PIP3 synthesis which is required for mTORC2 activation and Thr958 phosphorylation in the turn phosphate motif.

PKNs can be activated by growth factor receptors signaling through different pathways. TKR-induced PI3K activity catalyzes PIP3 synthesis which is required for mTORC2 activation and Thr958 phosphorylation in the turn phosphate motif. Furthermore, phospho-tyrosine residues of TKRs provide docking sites for SH2 and PTB domains. NCK adapter protein binds phosphorylated Epidermal Growth Factor Receptor (EGFR), Platelet-Derived Growth Factor Receptor (PDGFR), Vascular Endothelial Growth Factor Receptor (VEGFR) via its SH2 domain and is a mediator of growth factor-induced signal transduction [69,70,71]. PKN2 specifically binds both the SH3 domains of NCK and Rho in a GTP-dependent manner, cooperating with Rho family proteins to induce transcriptional activation via the serum response factor [69]. Therefore, PKN2 may coordinate signal transduction from activated TKRs to Rho, and NCK may act as an adapter molecule connecting receptor-mediated events to Rho protein signaling [69]. Therefore, PKN2 is activated by Rho-dependent PDK1 phosphorylation in the activation loop and by mTORC2-dependent phosphorylation in the turn phosphate motif. Our phospho-proteomic analysis shows that PKN2 is phosphorylated at Thr958 residue upon FPR2 stimulation [38], suggesting the involvement of the FPR2-induced signaling in the regulation of this phosphorylation. We hypothesize two possible molecular mechanisms of activation. Binding WKYMVm/FPR2 activates Rho which induces a conformational change in PKN2 allowing the binding and the consequent phosphorylation by PDK1 in the activation loop. mTORC2, activated by FPR2 signaling, phosphorylates the Thr958 residue in the turn phosphate motif (Figure 1a). Our phosphoproteomic analysis also reveals a pivotal role played by β-arrestin-1 [38] which is involved in FPR2 internalization and/or formation of FPR2-dependent signaling scaffolds [72,73,74]. In fact, β-arrestin isoforms modulate RhoA activity, affecting actin cytoskeletal organization, cell proliferation and cell cycle progression [75]. Therefore, we cannot exclude a β-arrestins-dependent signaling involved in RhoA-mediated PKN2 activation (Figure 1a). We previously demonstrated the FPR2-mediated EGFR transactivation [26]. Phospho-tyrosines of transactivated EGFR can bind the SH2 domain of NCK and PKN2 associates to the adapter protein via the SH3 domain. The subsequent binding to Rho induces a conformational change in PKN2, which is required for PDK1 phosphorylation in the activation loop of PKN2. Furthermore, EGFR-induced PI3K activity triggers PIP3 synthesis which represents the prerequisite for mTORC2 activation and Thr958 phosphorylation in the turn phosphate motif of PKN2 [58] (Figure 1b). Consistently, phosphorylation of this residue has been also observed in Epidermal Growth Factor (EGF)-stimulated HeLa cells [76], as well as in the kinome analysis during cell cycle [77,78,79], and in human cancer cells phospho-proteome [80].

PKN2 is one the first identified effector proteins of Rho [69] and plays critical roles in actin cytoskeletal organization [81], in the apical junction formation [45], in controlling cell-cell adhesion of keratinocytes [82], invasion and migration of cancer cells [83,84]. Furthermore, the observation that PKN2 can phosphorylate, and thereby regulate, several cytoskeletal substrates [81] enhancing actin polymerization and contributing to tumor cell migration [85], strongly suggests that FPR2 signaling plays a key role in these molecular mechanisms.

## 3. Serine/Threonine-Protein Kinase PRP4 Homolog (PRP4; UniProt: Q13523)

PRP4 was originally isolated from *Schizosaccharomyces pombe*, but its expression has been subsequently observed in a variety of human tissues [86]. Human PRP4 encodes a 1007-amino acid protein containing an extended NH_2_-terminus, which is characterized by basic aminoacids repeats, able to act as a NLS, and Arg/Ser (RS) domains, frequently found in pre-mRNA splicing factors whose deletion induces the cytosolic localization of the protein [86]. PRP4 belongs to a family of Ser/Arg (SR)-rich protein-specific kinases able to recognize SR-rich substrates [86,87] and, based on kinase domain sequence homology, it is classified as a member of CMGC group. This includes the **C**dk, **M**APK, **G**SK, and cdc2-like kinase (**C**lk) protein families [88]. PRP4 belongs to the Clk family [88]. MAPK family includes extracellular signal-regulated kinases (ERKs), c-Jun N-terminal kinases (JNKs), and p38MAPKs, which are activated by the dual Tyr/Thr phosphorylation of a conserved TEY, TPY or TGY motif, respectively [89]. The region between the kinase subdomains VII and VIII, within the catalytic domain of PRP4, shows significant sequence homology to MAPKs [87,90,91]. Thr336 and Tyr338 residues located in in this region are in an equivalent position in the TEY, TPY, and TGY sequences of the other known MAPKs [87], whereas Thr847 and Tyr849 residues in the subdomain VIII of PRP4 are in a similar position in the TPY sequence of JNK/SAPK [87]. The phospho-proteomic analysis we carried out revealed that Tyr849 residue of PRP4 is phosphorylated in FPR2-induced signaling [38].

**Figure 2:**
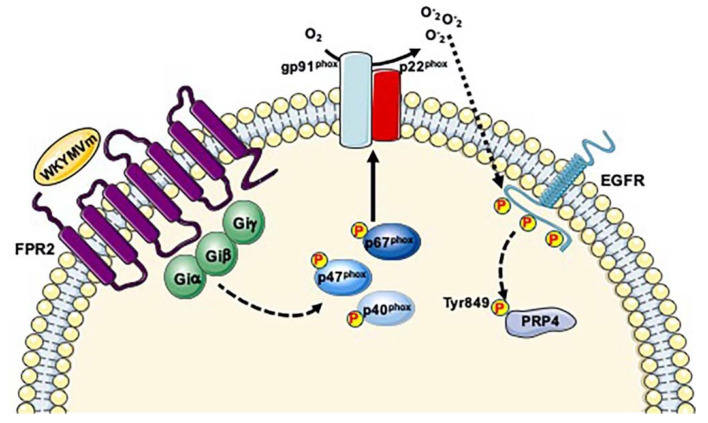
FPR2-mediated EGFR transactivation induces Tyr849 phosphorylation of PRP4. FPR2 signaling induces the phosphorylation of cytosolic regulatory subunits of NADPH oxidase and, in turn, ROS generation which bridge the signals from FPR2 to EGFR. Phospho-tyrosine residues of EGFR provide docking sites for recruitment and triggering of not yet identified kinases, which in turn phosphorylate PRP4 at Tyr849 residue.

PRP4 is involved in pre-mRNA splicing. In fact, it is able to phosphorylate the RS domain of the mammalian splicing factor ASF/SF2 [87], which belongs to the SR superfamily of splicing factors (SRSFs). SRSFs participate in constitutive or in alternative splicing and they act as chaperones to couple splicing with transcription and RNA nuclear export. Reversible phosphorylation of SR proteins modulates their versatile functions [92] and phosphorylation of RS sites is crucial for pre-mRNA splicing and spliceosome assembly. Interestingly, PRP4B, an isoform of PRP4, is a component of U4/U6 snRNP and the pre-mRNA processing factors PRP6 and PRP31, both components of the U4/U6-U5 snRNP, are directly phosphorylated by PRP4 concurrently with their incorporation into spliceosomal B complexes [93]. An aberrant PRP4 kinase activity or its loss of function leads to the accumulation of pre-mRNAs, which perturbs mitosis and subsequently induces the elongation of the cell cycle [94]. About 77% of phospho-proteins identified in our phospho-proteomic analysis are RNA-binding proteins [38] and, coherently, we observed PRP4 phosphorylation at Tyr849 residue in WKYMVm-stimulated cells, suggesting that FPR2 signaling triggers transcription and/or splicing events. Phosphorylation at Tyr849 residue of PRP4 has been reported during mitotic phase of the cell cycle [78,79], in human embryonic stem cell differentiation [95] and in phospho-tyrosine profiling of NSCLC cells in response to EGF and HGF [96]. PRP4 also phosphorylates Thr residues in ElkC suggesting that it plays a role in signal transduction in addition to mRNA processing [90]. However, the dual phosphorylation of the T^847^PY^849^ motif in the subdomain VIII of PRP4 is not essential for kinase activity which requires, at least partially, the autophosphorylation on Ser and Tyr residues [90]. Nevertheless, EGF stimulates PRP4 even though it is not clear what kinase, if any, is upstream to PRP4 [90]. PRP4 is a HER2/ERBB2-regulated gene in breast and ovarian cancer [97] and plays a crucial role in regulating the endosomal trafficking of EGFR leading to altered anoikis sensitivity. In fact, PRP4 depletion reduces EGF-stimulated EGFR degradation and promotes sustained growth factor signaling [97]. Previously, we demonstrated that FPR2 stimulation by WKYMVm induces ROS-dependent EGFR transactivation and that phospho-tyrosine residues provide docking sites for recruitment and triggering of several kinases [26]. We speculate that intracellular signaling cascades elicited by FPR2-mediated EGFR trans-phosphorylation could mediate the phosphorylation at Tyr849 residue of PRP4 (Figure 2). Despite this phospho-site may not be an activating residue, it could impair PRP4 kinase activity or its interaction with regulatory proteins.

## 4. Serine/Threonine-Protein Kinase MARK2 (MARK2; Uniprot: Q7KZI7)

MAP-microtubule affinity-regulating kinases (MARKs) were discovered for their ability to phosphorylate tau and related microtubule-associated proteins (MAPs) on Ser residues located within the KXGS motifs. Phosphorylation of tau results in the detachment from microtubules and in the impairment of microtubule-based intracellular transport [98,99]. In C. elegans the orthologues of human MARK is Par-1, which belongs to a family of partitioning-defective (PAR) gene products crucially involved in the establishment of cell polarity [100] and highly conserved from yeast to human [101,102,103]. The 5′AMP-activated protein kinase (AMPK) and Par-1 family members are closely related and show 50% identity across their kinase domains, as well as similar phosphorylation site preferences. Par-1 and AMPK share a common consensus phosphorylation motif and can phosphorylate a Ser residue when Leu/Ile/Met, Arg/Lys, and Leu are present in the −5, −3 and +4 positions, respectively [101].

The alpha subunits of AMPK belong to a family of 12 related kinases (AMPK-related kinases; ARKs), namely BRSK1, BRSK2, PAR-1c/MARK1, PAR-1b/MARK2, PAR-1a/MARK3, PAR-1d/MARK4, MELK, NUAK1/ARK5, NUAK2/SNARK, SIK1, SIK2/QIK and SIK3/QSK [104]. PAR-1c/MARK1, PAR-1b/MARK2, PAR-1a/MARK3, PAR-1d/MARK4 form a subfamily of calcium/calmodulin-dependent protein kinases (CAMK) closely related to the AMPK subfamily. All MARK isoforms show a similar organization and in their sequence it is possible recognize: (i) a NH_2_-terminal header, (ii) a catalytic kinase domain, (iii) a linker, (iv) a UBA domain, (v) a spacer, and (vi) a tail domain [105]. The role of the header is unknown and in several splice variants, it is missed or reduced in size. The kinase domain is linked to a small α-helical domain (UBA domain), which probably has autoregulatory functions. The most variable region, the spacer domain (290–330 residues), contains phosphorylation sites targeted by atypical PKC (aPKC) and/or protein kinases D and plays a crucial role in the regulation of MARKs activity. A possible autophosphorylation site in the spacer domain has been identified within the residues 627–636 in MARK2 [106].

Interestingly, both PAR-1b/MARK2 and AMPK are regulators of metabolism and show overlapping functions in vivo [101,107]. In general, they promote all processes involved in ATP generation, such as glycolysis, β-oxidation of fatty acids and oxidative phosphorylation, and prevents protein translation and lipid synthesis processes, which consume ATP. Constitutive deletion of MARK2, 3 or 4 results in enhanced insulin sensitivity, resistance to high-fat-diet-induced obesity and hypermetabolic phenotypes of varying severity, suggesting that these kinases play a key role in the development of diabetes [108,109] and that FPR2 signaling is involved in the regulation of metabolism. PAR-1b/MARK2 is also implicated in several others physiological processes, including fertility, immune system homeostasis, learning, memory, and growth [108,110,111,112].

In addition to tau and MAPs, MARKs phosphorylate several substrates in their binding sites, such as mitogen-activated protein kinase scaffolding protein KSR1, tyrosine phosphatase PTPH1, cell cycle-regulating phosphatase Cdc25, class IIa histone deacetylases and plakophilin. Such phosphorylations enhance the binding of these proteins to 14-3-3 modifying their subcellular localization and affecting regulatory pathways [113,114].

MARK2 is activated by phosphorylation of a conserved Thr208 in the activation loop (T loop) located within the catalytic domain. MARKK/TAO-1 and the tumor suppressor Liver kinase B1 (LKB1) in complex with STRAD and MO25 phosphorylate MARK2 at this site [115,116]. On the other hand, phosphorylation of MARK2 by GSK3β on a Ser212 residue located within the T loop, close to the primary phosphorylation residue, prevents MARK2 kinase activity [117]. Phosphorylation inside or outside the catalytic domain directly affects the kinase activity or mediates the interaction with regulatory proteins. CaMKI phosphorylates MARK2 at a Thr294 residue, but only if it is activated by calcium and calmodulin [118]. aPKCs phosphorylate MARK2 at Thr595 residue, which is located in the spacer in the COOH-terminal domain. Phosphorylation at this site stimulates the spatial redistribution of MARK2 from cell membranes to the cytoplasm by binding to 14-3-3 [119,120,121]. PAK5, a member of the p21-activated kinase family, suppresses the activity of MARK2 and this inhibition does not require phosphorylation but rather the binding between the PAK5 and MARK2 catalytic domains [122]. Autoregulation by the accessory UBA domain (inhibitory or stimulating) is still a matter of debate. Although formation of dimers is a common mechanism of regulation observed for many kinases, autoinhibition of MARK2 by dimerization lacks of in vivo evidence [105]. Therefore, MARK2 activity is triggered by MARKK/TAO-1 and LKB1, and inhibited by GSK3β, aPKCs and PAK5.

**Figure 3:**
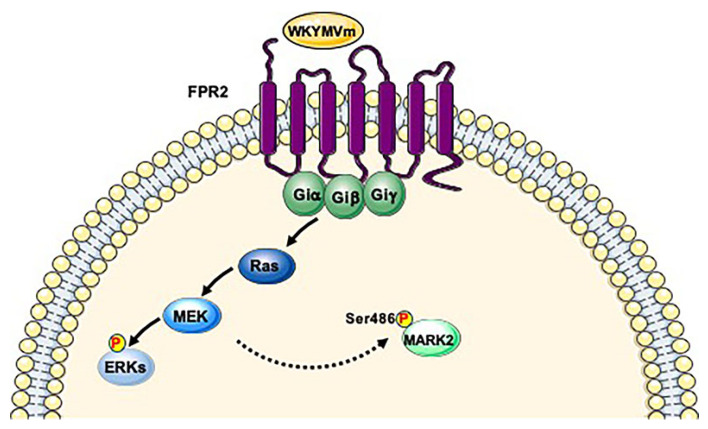
FPR2 signaling triggers MARK2 phosphorylation. FPR2 stimulation triggers Ras/MAPK pathway. We hypothesize that Ser486 residue of MARK2 is phosphorylated by not yet identified kinases downstream to Ras cascade.

MARKK/TAO-1 is regulated by Ras GTPases family [115,123] and Ras/ERK pathway affects LKB1 function via ERK-mediated Ser325 phosphorylation and p90RSK-mediated Ser428 phosphorylation [124]. Our phospho-proteomic analysis revealed FPR2-dependent Ser486 phosphorylation of MARK2 [38], which has never been described as a residue phosphorylated by MARKK/TAO-1 and/or LKB1. FPR2 signaling does not elicit GSK3β, aPKCs and PAK5 pathways and other PKs able to phosphorylate the Ser486 of MARK2 have not been identified. Since FPR2 stimulation triggers G proteins dissociation and Ras/ERK pathway [15,21,22,24,26], we hypothesize that Ser486 residue of MARK2 is phosphorylated by not yet identified PKs downstream to Ras cascade. (Figure 3). This phospho-site has been also observed, both in MARK2 and AMPK, in cell cycle progression [77], in human kinome analysis [125] and in human embryonic stem cell differentiation [95]. Although it has not been demonstrated that the Ser486 of MARK2 is an activating residue, it could directly affect MARK2 activity or could represent a docking site for other proteins.

## 5. Serine/Threonine-Protein Kinase PAK4 (PAK4; Uniprot: O96013)

The p21-activated kinases (PAKs) belong to the Ser/Thr kinases family which is composed of six members distributed into a group I (PAK1, PAK2, and PAK3) and a group II (PAK4, PAK5, and PAK6). The PAK family proteins show a similar structure that can be divided into three main domains: an NH_2_-terminal PBD (p21-GTPase-binding domain), also known as a Cdc42 Rac Interactive Binding (CRIB) domain [126], a central region and a highly conserved COOH-terminal serine/threonine kinase domain [127]. Even though PAK4, PAK5, and PAK6 show a short NH_2_-terminal PBD sequence, there is considerable homology within the PBD between all PAK family members. PAK1-3 bind to Rac and Cdc42, whereas PAK4-6 binds preferentially Cdc42. None of the PAK family members bind to the Rho isoforms RhoA, RhoB, and RhoC [128]. The central regions of PAK4, PAK5 and PAK6 contain several proline-rich potential SH3-domain-binding sites. A RhoA GEF-binding site has been identified in the central region of PAK4, but not in PAK5 and PAK6 [129], and an integrin-binding domain within the kinase domain has been reported for PAK4 [130].

The activation mechanisms of groups I and II of PAKs are different and the relative regulatory domains are structurally distinct [131]. The kinase activity of group I of PAKs is stimulated when Rac or Cdc42 binds to PBD, whereas in PAK4, PAK5, and PAK6 it is independent on Cdc42 activation [128], suggesting that in group II PAKs the kinase is constitutively active [132,133]. However, group II PAKs can be activated by signaling cascades downstream of growth factor receptors, in particular by Hepatocyte Growth Factor (HGF)/cMet binding, through a PI3K-dependent signaling [134,135,136,137], but the molecular mechanism of kinase activation is still unclear [134,135]. NH_2_-terminal regions of PAK4 and PAK5 can interfere with kinase activity. In fact, removal of the PBD enhances PAK4 and PAK5 kinase activity [135,138], suggesting a regulatory mechanism similar to that identified for group I PAKs. However, an NH_2_-terminal fragment able to inhibit kinase activity has not been identified in PAK4. The kinase activity of PAK4 can be inhibited by the PI3K inhibitor LY294002 but the mechanism of regulation of PAK4 kinase activity by PI3K is still unclear [127,135]. PAK4 has been localized in the perinuclear region and at the cell periphery downstream of growth factor-mediated signaling [135] suggesting that it may shuttle between cytoplasmic compartments. PAK4 also contains an NLS, mapped to a polylysine sequence (KKKK) in the NH_2_-terminal region, and a nuclear export sequence [127,132].

Therefore, PAK4 is mainly activated by cMet-induced PI3K signaling. Noteworthy, PAK4 and the p85 α-subunit of PI3K specifically interact [135] and the development of specific inhibitors able to dissociate PI3K from PAK4 represent a novel therapeutic approach in several types of cancers in which PI3K and PAK4 play a central role in tumor progression [139]. FPRs stimulation induces PI3K activity [140] and the conversion of phosphatidylinositol 3,4-diphosphate to PIP3 by PI3K leads, among other things, to the activation of Rho GTPase family members, such as Rac1 and Cdc42 [141,142].

**Figure 4:**
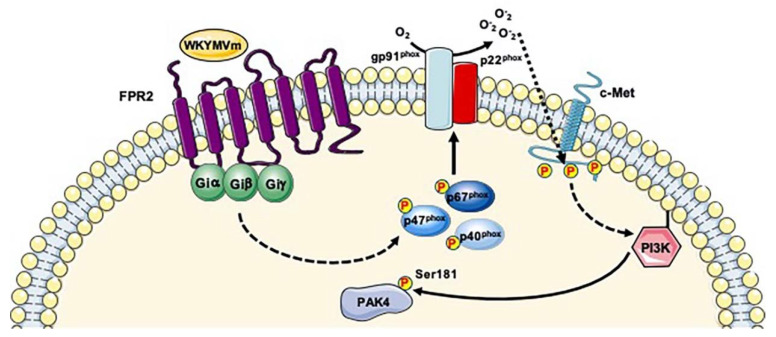
FPR2 signaling induces PI3K-mediated Ser181 phosphorylation of PAK4. Binding of WKYMVm to FPR2 induces NADPH oxidase-dependent c-Met transactivation. Phospho-tyrosine residues of c-Met trigger PI3K signaling that promote PAK4 phosphorylation.

Previously, we showed that FPR2 stimulation by WKYMVm induces the trans-phosphorylation of Y1313/Y1349/Y1356 residues of c-Met and elicits some of the molecular responses triggered by c-Met/HGF binding, such as STAT3, phospholipase C (PLC)-γ1/PKCα and PI3K/Akt pathways [25,143]. We also demonstrated that blockade of NADPH oxidase function prevents c-Met transactivation and the downstream signaling cascades, highlighting the critical role of NADPH oxidase-dependent ROS generation in this molecular mechanism [25]. We hypothesize that FPR2- and NADPH oxidase-mediated c-Met trans-phosphorylation may induce PI3K signaling thus promoting PAK4 activation (Figure 4). We observed the phosphorylation of PAK4 at Ser181 residue in FPR2-stimulated cells [38] which represents, in addition to phospho-Ser99, the major site responsible for the binding of PAK4 to 14-3-3 [144]. Since 14-3-3 is a multi-functional scaffold protein, it may link PAK4 to several cellular functions. In fact, PAK4 regulates cytoskeleton remodeling, gene expression, directional motility, invasion, metastasis, and growth [145] by phosphorylation of several substrates such as LIMK1, integrin β5, p120-catenin, SCG10, β-catenin and Smad2 [129,134,146,147,148,149,150,151]. These observations strongly suggest that FPR2 signaling is involved in the modulation of many cellular functions by regulating PAK4 phosphorylation at Ser181 residue and, in turn, the binding of PAK4 to 14-3-3. The phosphorylation of Ser181 residue of PAK4 was observed in mitosis [77,78], in human kinome analysis [125], in TCR signaling analysis [152], in human embryonic stem cells differentiation [95], in human cancer cells and human liver phospho-proteomic analysis [80,153], as well as in the analysis of 14-3-3 interactome [144].

## 6. Serine/Threonine-Protein Kinase 10 (STK10; UniProt: O94804)

In mice, lymphocyte-oriented kinase (LOK) is a member of the STE20 family characterized by serine/threonine kinase activity, whose expression is restricted mostly to lymphoid cells [154]. It consists of 966 amino acids with a kinase domain at the NH_2_-terminus, which shows similarity to that of the STE20 family of serine/threonine kinases involved in regulating MAPK cascades, and a long coiled-coil structure at the COOH-terminus. The two domains are separated by a proline-rich region. In yeast STE20 is a component of the MAP kinase cascade required to transmit the pheromone signal from Gβγ subunits to downstream components [155]. STE20 phosphorylates STE11 that phosphorylates STE7, thereby acting as a MAPKKK. STE7 in turn phosphorylates FUS3 and KSS1 MAP kinases, thus functioning as a MAPKK [156]. Several mammalian homologs of STE20 are involved in the MAPK cascades activated by extracellular stimuli such as mitogens, cellular stresses, and cytokines [157,158].

STK10 is the human homolog of LOK. It shows high similarity to LOK with a 98% and 93% of identity in the kinase domains and in the coiled-coil regions, respectively [159]. STK10 shows high similarity also to members of Polo-like Kinase Kinase (Plkk) family, such as the human Ste20-like kinase (SLK) and X. laevis Plkk1, suggesting that it can exert control over the activity of Polo-like kinase 1 (Plk1) [160]. STK10 is expressed in various tumor cell lines, as well as in highly proliferative normal tissue, where it co-associates with and phosphorylates Plk1. Plk1 activity is of critical importance to successful cell division, and cells expressing an STK10 inactive mutant show an altered cell cycle phenotype [160].

STK10 is a key kinase for tumor progression [161] and siRNA knockdown of STK10 increases apoptosis of tumor cells [162]. Mutations in the kinase domain of STK10 have been identified in human testicular germ cell tumors, and mutations in the coiled-coil region have been described in aggressive lymphoma [163,164]. Interestingly, these mutations inhibit apoptosis, suggesting that STK10 may act as a tumor suppressor [164]. In contrast, in Ewing sarcoma cell lines, RNA interference of STK10 promotes cell survival and growth [162], suggesting that it might exert pro- or anti-apoptotic functions according to the cell type. FPR2 promotes cancer progression in several cell lines [165,166,167,168] and knocking down of FPR2 from colon cancer cell lines reduces their tumorigenicity [169]. FPR2-dependent STK10 phosphorylation could mediate cell proliferation and tumor progression.

STK10 phosphorylates ezrin, radixin and moesin (ERM), which mediate linkage of actin cytoskeleton to plasma membrane [161] and contribute to the maintenance of microvilli and to cell rigidity [170]. In epithelial cells STK10-mediated phosphorylation of ERM culminates in the phosphorylation at Thr567 residue of ezrin [171]. The ERM proteins share a common domain which contains a binding pocket for phosphatidylinositol 4,5-bisphosphate [PI(4,5)P2] [172]. Since STK10 and PI(4,5)P2 localization is enriched on the apical membrane and both are required for ezrin phosphorylation, ezrin activation only occurs on the apical domain [171,173].

**Figure 5:**
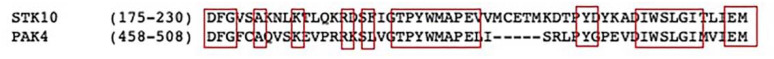
Structure-based sequence alignment of activation segment regions. The similar activation segments of STK10 and PAK4 are shown.

The upstream signaling cascades regulating STK10 kinase activity remain to be elucidated, although STK10 catalytic activity seems to depend on dimerization of the kinase domain and, in turn, on STK10 trans-phosphorylation [174]. Interestingly, PAK4 and STK10 share similar segment activation regions (Figure 5). A modified conformation of these segments results in an active kinase in trans, with the phosphorylation sites in proximity to the active site. This represents a common regulatory mechanism of several protein kinases [174].

We found that Ser438 and Thr952 residues of STK10 are phosphorylated in FPR2-stimulated cells [38]. Phosphorylation of Ser438 residue has been described in nocodazole-arrested HeLa cells [175], in human primary circulating T lymphocytes [176], in ADP-stimulated platelets [177,178], during cell cycle progression [77,79] and in different cancer cell lines [80,125]. Phosphorylation of Thr952 residue has been described during mitosis [78] and in human liver phospho-proteome [153].

## 7. Dual Specificity Mitogen-Activated Protein Kinase, Kinase 2 (MAP2K2; UniProt: P36507)

Dual specificity mitogen-activated protein kinase, kinase 2 (MAP2K2) is also known as CFC4, or MAPKK2, or MEK2 (Mitogen/Extracellular signal regulated kinase, kinase 2), or MKK2 (MAP kinase kinase 2), or PRKMK2. MEK1 and MEK2 are ubiquitously expressed and are encoded by two distinct genes located at different chromosomes [179]. The MAP2K1 gene is located on chromosome 15q22.31 and encodes for MEK1, whereas MAP2K2 gene is located on chromosome 19p13.3 and encodes MEK2 [180]. MEK1/2 trigger the activation of diverse cellular responses, many of which play a key role in tumorigenesis. In fact, the two kinases contribute to the nuclear events that regulate cell proliferation and differentiation, gene expression, cell cycle, embryogenesis, motility, metabolism, programmed cell death, and angiogenesis [179,181,182]. FPR2 stimulation promotes tumor cell invasion by evoking MEK/ERK pathway [183,184,185], which represents a common event in cancer progression. For instance, FPR2 activation enhances the invasion and metastasis of gastric cancer and astrocytoma cells by activating MEK/ERK pathway [185,186]. MEK1 and MEK2 share the same overall structure, namely an ERK docking region, a negative regulatory region, a Proline-rich insert within the kinase domain, a nuclear export sequence and a pocket structure adjacent to the ATP-binding site [187,188]. The nuclear export signal is required for cytoplasmic localization (and nuclear exclusion) of MEK1/2. The function of the COOH-terminal residues is still unclear. It could contain a recognition motif for the activating kinase of MEK1/2 or a cellular translocation signal [187]. Binding of specific inhibitors within the pocket structure triggers conformational changes that lock unphosphorylated MEK1/2 into a catalytically inactive state [189]. Since MEK1 and MEK2 are involved in about 20% of all cancers and more than 60% of melanomas, their inhibition is an attractive therapeutic strategy [190]. In fact, numerous specific MEK1/2 inhibitors have been developed and evaluated in several clinical studies [181]. However, MEK1 and MEK2 serve distinct biological functions that may be determined by specific modulation of their activity levels. For instance, MEK1 contains a phospho-site (Thr292), which is required for ERK-mediated feedback phosphorylation that is missing in MEK2. In addition, MEK2 knockout mice are viable and phenotypically normal, whereas knockout MEK1animals do not survive during embryogenesis [191]. MEK1/2 are expressed in neutrophils and both are required for ERKs and oxidative burst activation, but MEK2 represents the predominant functionally isoform in N-fMLP-treated polymorphonuclear leukocytes (PMN) [192]. In these cells MEK2 activity is at least 3-fold higher than that of MEK1 [192] and forms an inactive complex when associated with p38MAPK. This is dissociated and activated upon N-fMLP stimulation, through a pathway involving PI3K and PKC [193].

NADPH oxidase activation requires the phosphorylation of the cytosolic components p40^phox^, p47^phox^ and p67^phox^ and their translocation to the membrane, followed by their interaction with cytochrome b558 [21,22,23,24,25,26,194,195,196]. In FPRs-stimulated cells PKC- and ERK/p38MAPK-dependent pathways phosphorylate p67^phox^ on Ser and Thr residues [197,198,199] and the inhibition of MEK activity prevents p47^phox^ phosphorylation [200,201], suggesting that NADPH oxidase-dependent ROS generation requires MEK1/2 activity. Previously, we demonstrated that in WKYMVm-stimulated human fibroblasts FPR2 triggers MEK-dependent p47^phox^ phosphorylation and membrane translocation, as well as NADPH-dependent superoxide production [21,22].

**Figure 6:**
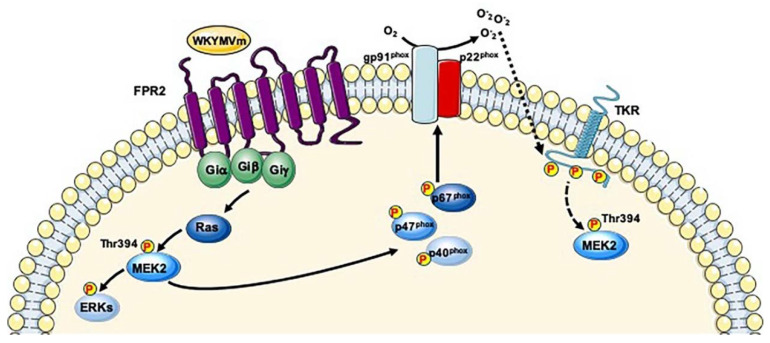
FPR2-mediated Thr394 phosphorylation of MEK2. Binding of WKYMVm to FPR2 triggers both Gi proteins-induced Ras/MEKs/ERKs signaling and ROS-dependent TKRs transactivation. Phosphorylated tyrosines of TKRs elicit Ras/MAPK pathway. Thr394 phosphorylation of MAP2K2 might depend on both signaling cascades.

In a typical signaling pathway triggered by TKRs, the phosphorylated tyrosine residues of the TKRs act as docking sites for several proteins, including Shc, Grb2 and SOS that link the activated TKR to Ras-signaling pathway. The GTP-bound Ras induces the translocation of the Ser/Thr kinase Raf-1 to the plasma membrane, where it is activated by multiple phosphorylation events [202,203]. Raf-1, in turn, phosphorylates MEK1 and MEK2 on Ser217/218 and Ser221 residues, respectively. MEK1 activates ERK1, whereas MEK2 activates ERK2 through phosphorylation of the specific Thr183 and Tyr185 residues located within the characteristic TPY motif. The Ras–Raf–MEK1/2–ERK1/2 pathway is considered the ‘classical MAPK pathway’ and is one of the most frequently impaired signaling cascades in human cancer. However, the Ras-Raf-MEK1/2-ERK1/2 signaling pathway is also triggered by GPCRs [204], but the mechanisms by which signals are transduced from receptor coupled to Gs, Gq and Gi proteins to the MEK/ERK cascade are not completely clear, even though the involvement of Gα and Gβγ subunits is implicated [205]. GPCRs can trigger Ras-Raf-MEK1/2-ERK1/2 cascade also through TKR transactivation [18] and we previously demonstrated that FPRs stimulation induces NADPH oxidase-dependent TrkA, VEGFR2, HGF, and EGFR trans-phosphorylation. Phosphorylated tyrosines of these TKRs provide docking sites for signaling molecules that activate, among other things, Ras-Raf-MEK1/2-ERK1/2 pathway [23,24,25,26]. In the phospho-proteomic analysis of FPR2-stimulated human lung cancer cells we observed the phosphorylation at Thr394 residue of MAP2K2 [38], located in the COOH-terminal tail. The role of this phospho-site is matter of debate. It was observed by analyzing phosphorylation sites in mitosis [78,79], in embryonic stem cell differentiation [95], in human cancer cells [80], in 2-Deoxyglucose-stimulated cells [206], as well as in rat models of drug-induced kidney injury caused by cisplatin and puromycin [207]. The Thr394 residue is located within the sequence 389-LNQPGTPTRI-397 in the COOH-terminal tail which could represent a recognition motif for the kinase upstream MEK2 or a cellular translocation signal [187]. We propose that both Gi proteins-induced signaling and FPR2-mediated TKRs transactivation can mediate Thr394 phosphorylation of MAP2K2 (Figure 6). Furthermore, we suggest that FPR2-induced MEK2 phosphorylation promotes tumor cell invasion by evoking MEK/ERK pathway.

## 8. Protein Phosphatase 1 Regulatory Subunit 14A (PPP1R14A; UniProt: Q96A00)

PPP1R14A is also known as CPI-17, CPI17 or PPP1NL. Its structure shows intrinsically disordered NH_2_- and COOH-terminal tails and a central PHIN domain that consists of 86 residues [208,209,210]. The PPP1R14A gene is located on chromosome 19 and encodes a 147-residue polypeptide in which more than 85% of the amino acids are highly conserved within mammals [210,211]. Three CPI-17 homologs, namely Phosphatase Holoenzyme Inhibitor (PHI), Kinase Enhanced Phosphatase Inhibitor (KEPI), and Gastrointestinal and Brain-specific PP1-Inhibitory protein (GBPI) have been identified in humans, which show a PHIN domain homologous more than 41% to CPI-17 [210] and a RVXF motif in the NH_2_-terminal tail domain, absent in CPI-17 [212].

CPI-17 is expressed predominantly in smooth muscles where it plays crucial regulatory functions [210,213,214]. In fact, Myosin Light Chain Phosphatase (MLCP) activity, a master regulator of smooth muscle responsiveness to stimuli, is regulated by CPI-17 and by Myosin Phosphatase Target subunit 1 (MYPT1) regulatory subunit. The regulation of CPI-17 expression is partly mediated by kinase-mediated signaling. In fact, PDGFR, a potent smooth muscle growth factor, activates ERKs that prevent Sp1/Sp3 binding to the CPI-17 gene promoter, inhibiting its transcription. On the other hand, PKC and ROCK positively regulate CPI-17 promoter by sequential phosphorylation [215].

A major kinase for GPCR-induced CPI-17 phosphorylation is PKC which is activated by the PLCβ-produced signaling messenger diacylglycerol (DAG). It phosphorylates CPI-17 at Thr38 residue that directly docks at the active site of MLCP, thereby inhibiting its activity and promoting an increase of phosphorylation of myosin and of other MLCP substrates [213,216,217,218,219,220,221]. Therefore, a potential signaling pathway leading to MLCP inhibition is G protein/PLCβ/DAG/PKC/CPI-17/MLCP. CPI-17 can be also directly phosphorylated at Thr38 residue by MYPT1-associated kinase [222], by PAK, which is downstream of Rac and/or Cdc42 cascade [223], by Rho-associated coiled-coil kinase (ROCK) [224] and by PKN [225]. The observation that ROCK and PKN are the downstream protein kinases of Rho pathway suggests that CPI-17 activity may be controlled by Rho in addition to PKC signaling. Furthermore, the presence of multiple kinases that regulate CPI-17 suggests that this phosphatase plays the role of hub of signals that control MLCP activity. PKC and ROCK play distinguishable roles in CPI-17 phosphorylation. PKC induces the rapid phosphorylation of CPI-17 at Thr38 residue concurrent with a rapid rise in Ca^2+^ and an initial rapid MLCP phosphorylation. On the other hand, ROCK stimulates the Ca^2+^-independent phosphorylation of CPI-17 in the subsequent sustained phase, in parallel with a force generation [226]. CPI-17 functions are regulated not only by phosphorylation and expression, but also by cellular diffusion [216]. CPI-17 is distributed in the cytoplasm of mature smooth muscle cells but it accumulates also in nuclei under growth conditions, in human atherosclerotic plaques, and in a subset of cancer cells [227]. Sequence analysis of CPI-17 shows the presence of an unconventional NLS (1-MAAQRLGKRVLSKLQSPSRARGPGG-25) in the NH_2_-terminal tail [227]. In addition to the Thr38 residue, PKC and MYPT1-associated kinase phosphorylates the Ser12 residue within the NLS [222,228,229], and Ca^2+^/calmodulin-dependent protein kinase II phosphorylates the Ser130 residue at the COOH-terminal tail of CPI-17 [228,229]. The substitution of Ser12 with a phospho-Asp residue interferes with nuclear accumulation of CPI-17 in proliferating mature smooth muscle cells [227].

We observed that FPR2 stimulation induces the phosphorylation of Ser16 and Ser26 residues of CPI-17 [38]. Phospho-Ser16 is located within the NLS and may play an inhibitory role in the nuclear import. It is possible that pathological stresses of smooth muscle cells alter the phosphorylation state of NLS, regulating the subcellular distribution of CPI-17 [227]. FPR2 also contains an NLS resulting functionally expressed onto nuclear membrane, since its stimulation triggers the phosphorylation of nuclear ERK2, c-Jun and c-Myc [15].

**Figure 7:**
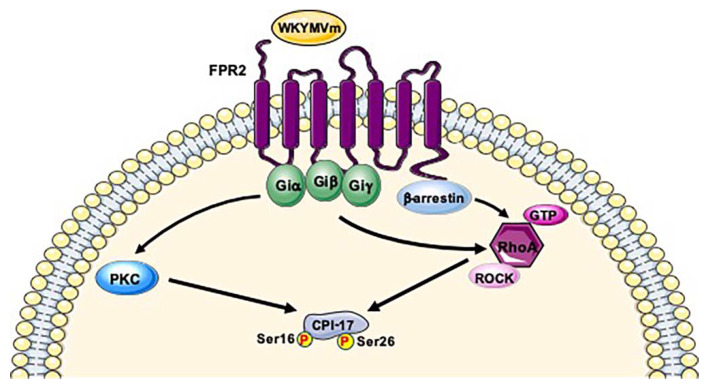
Phosphorylation of Ser16 and Ser26 of CPI-17 depends on PKC and ROCK pathway. FPR2 signaling induces PKC- and/or ROCK activation which mediate Ser16 and Ser26 phosphorylation of CPI-17. RhoA-ROCK cascade could be also activated by β-arrestin-induced signaling.

Therefore, CPI-17 activity is regulated by PKC and ROCK and several GPCR agonists induce PKC- and ROCK-dependent phosphorylation of CPI-17 at Thr38 residue [218,226,230]. FPR2 stimulation triggers PKC activation [196] and Rho-dependent chemotaxis [231]. Noteworthy, RhoA/ROCK pathway plays a crucial role FPRs-regulated NADPH oxidase activity [232]. However, since RhoA/ROCK pathway can be triggered also by β-arrestins signaling [233], and in our phosphoproteomic analysis many of uniquely phosphorylated proteins converge on β-arrestin-1 [38], we cannot exclude that a biased signaling linked to β-arrestins could be involved in CPI-17 phosphorylation (Figure 7). We speculate that PKC activation and/or RhoA-ROCK cascade triggered by FPR2 signaling may phosphorylate Ser16 and Ser26 residues of CPI-17 (Figure 7). Phosphorylation of the Ser16 residue has been also observed in breast and ovarian cancer [234,235]. Ser26 is the first aminoacid outside the NLS. Phospho-Ser26 has been identified in resting human platelets [177] and in human cancer cells [80]. Alterations in CPI-17 signals occur under several pathological conditions, such as hypertension, asthma, inflammation, and diabetes [236,237,238,239,240,241]. For instance, CPI-17 phosphorylation is increased in hypoxia-induced pulmonary hypertension [236], in airway smooth muscle during inflammation and in diabetic bladder smooth muscle [237,241]. On the other hand, CPI-17 is down-regulated during inflammation in intestinal smooth muscle [239]. This bidirectional regulation of CPI-17 in different smooth muscle tissues in response to inflammatory signals remains unknown. Since FPR2 signaling is also involved in inflammatory and anti-inflammatory responses, modulation of CPI-17 phosphorylation by FPR2 can provide new insights in the regulation of smooth muscle contraction.

## 9. Concluding Remarks

FPR2 is a versatile transmembrane protein activated by an array of agonists, which include structurally unrelated lipids and peptide ligands, resulting in different intracellular responses. The ability of FPR2 to mediate a multitude of physio-pathological processes can be traced to the different receptor domains used by distinct agonists. FPR2 represents a promising therapeutic target in several human diseases, but further studies are required to fully understand molecular mechanisms responsible for PKs and PPs activation upon stimulation with different agonists.

PKs mediate a network of highly complex signals. The understanding of the regulatory functions of kinases may represents a valuable tool to identify more effective therapies against several human diseases. In fact, many drug kinase inhibitors are used in the treatment of different types of cancer and phospho-proteomics represents an important analytical strategy to identify new phospho-sites and phospho-proteins, and in turn to develop new drugs in tumor biology.

Many proteins are phosphorylated, even though phosphorylation is only one of several types of reversible covalent PTMs, which also include methylation, acetylation, sumoylation, and ubiquitination. In phospho-proteins, only a limited subset of phosphorylated sites is regulated. Furthermore, the observation that single phospho-sites are differently regulated suggests that proteins function as platforms to integrate multiple incoming stimuli. This integration of signals could act in an independent- or dependent manner. In the first case, the phosphorylation of each site occurs independently from the others, whereas in the second case a “priming site” is necessary for additional phosphorylation events. Inactivating mutations of PKs have been identified only in a small number of human cancers, indicating that modifications in kinase activity by phosphorylation are among the main causes of disease progression, and that phospho-proteomic screens could be useful to monitor cancer development and to identify new potential therapeutic targets.
